# Atypical development of sequential manual motor planning and visuomotor integration in children with autism at early school-age: A longitudinal kinematic study

**DOI:** 10.1177/13623613241311333

**Published:** 2025-01-06

**Authors:** Anna Bäckström, Anna-Maria Johansson, Thomas Rudolfsson, Louise Rönnqvist, Claes von Hofsten, Kerstin Rosander, Erik Domellöf

**Affiliations:** 1 Department of Psychology, Umeå University, Sweden; 2 Department of Health, Education and Technology, Luleå University of Technology, Sweden; 3 Department of Occupational Health, Psychology and Sports Sciences, University of Gävle, Sweden; 4 Department of Psychology, Uppsala University, Sweden

**Keywords:** autism spectrum disorders, development, kinematics, longitudinal, motor planning, school-age children, visuomotor integration

## Abstract

Sensorimotor difficulties are common in children with autism spectrum disorder, and it has been suggested that motor planning problems underlie their atypical movements. At early school-age, motor planning development typically involves changes in visuomotor integration, a function known to be affected in autism spectrum disorder. However, there is a lack of detailed characterization of typical motor planning development during this stage, and how motor planning develops in children with autism spectrum disorder is largely unknown. This longitudinal kinematic study examined goal-directed sequential manual movements in children with autism spectrum disorder and in typically developing children across ages 7, 8, and 9 years. We manipulated goal-difficulty and availability of initial visual information to investigate visuomotor integration and chaining of subparts during movement performance. The results revealed emerging group differences at older age, suggesting atypical motor planning development in children with autism spectrum disorder. Notably, unlike the typically developing group, availability of initial visual information did not facilitate motor planning for the autism spectrum disorder group. The results show that motor planning differences in autism spectrum disorder appear related to atypical visuomotor integration and global processing of sensorimotor information. The findings also emphasize the importance of considering developmental aspects in research and practice related to motor problems in children with autism spectrum disorder.

**Lay abstract:**

Many children with autism struggle with movement difficulties, yet the causes of these difficulties remain unclear. One possible explanation is atypical motor planning and integration of visual and motoric information. Before performing a goal-directed movement, the brain creates a prediction of the movement based on visual and sensory information and previous experience, forming a “blueprint” of the motor steps needed to achieve the goal. This process is called motor planning. During movement, adjustments to the plan can be made through feedback mechanisms. This longitudinal study aimed to examine the development of motor planning in children with autism and typically developing children over early school-age (7–9 years). The children performed a sequential manual peg-rotation task, which involved grasping, rotating, and placing a peg, while detailed measures of movement were collected. Task end-goal difficulty varied, and the goal was either initially occluded or fully visible. The results revealed that children with autism showed atypical motor planning development compared with typically developing peers, and these differences became more pronounced as the children grew older. As the typically developing children matured, they appeared to rely more on initial visual information, which assisted them in motor planning. However, this facilitation did not occur for children with autism. These findings suggest that the differences in motor planning seen in children with autism may be linked to atypical visuomotor integration, highlighting the need for individualized interventions. Furthermore, it is crucial to consider developmental aspects to fully understand motor planning in children with autism.

## Introduction

Every day, we execute numerous goal-directed manual activities, and the rapid generation of efficient movements is important to meet the demands of the environment. For children with autism spectrum disorder (ASD), atypical movements are frequently described but the etiology remains unclear (Zampella et al., 2021). One proposed mechanism for atypical movements in children with ASD is motor planning deficits (Martel et al., 2023; Von Hofsten & Rosander, 2012). Efficient motor performance is supported by motor planning, a process containing the outcome prediction of an action goal and the organization of motor behaviors needed to realize this goal. Action predictions require the integration of existing multisensory information with already formed action representations (Wong et al., 2015). This prediction is used to organize the required motor behaviors to execute efficient actions. Efficient motor planning hence relies on several functions, such as multisensory integration and coherent planning of sequences, known to be affected in ASD (Robertson & Baron-Cohen, 2017; Sinha et al., 2014). During action, feedforward mechanisms of the motor plan are supported by feedback mechanisms and, if needed, corrections of the initial motor plan according to sensory feedback.

It has been suggested that sensorimotor processes in motor planning may operate differently in ASD due to delayed multisensory development (Shafer et al., 2021), more taxing visual-proprioceptive integration (Glazebrook et al., 2009), and less reliance on visual information (Dowd et al., 2012; Lidstone & Mostofsky, 2021) in both children and adults with ASD. It has further been suggested that children with ASD rely less on feedforward processes and consequently show less chaining of sequential movements (Bäckström et al., 2021; Cavallo et al., 2018; Fabbri-Destro et al., 2009). Most everyday manual actions are sequential. For example, when reaching to grasp an object, we do so in order to use it. However, without chaining, which means making movement adjustments during a preceding motor act to accommodate the final goal of a subsequent motor act, movement efficiency is negatively affected.

Although motor planning difficulties constitute an important part of the movement problems observed in ASD, findings are inconsistent in describing a common characterization of disturbances. A plausible explanation for these inconsistencies could be related to developmental aspects. Previous cross-sectional studies have shown that motor planning kinematics are affected in dynamic ways dependent on task difficulty and child maturation. This has been observed both in typically developing (TD) children (Martel et al., 2020) and in children with ASD (Rodgers et al., 2019). Atypical motor planning seems to generate more pronounced effects on performance in older children with ASD compared with younger children with ASD (Rodgers et al., 2019). These dynamic effects might relate to younger children in general being more prone to exploratory strategies based on feedback processing than older children (Kuhtz-Buschbeck et al., 1998; Martel et al., 2020; Olivier et al., 2007). There have also been descriptions of increasing atypicality in manual motor performance over school-age years in children with ASD (Travers et al., 2017).

Regarding the typical progression of sequential actions, developmental changes in motor planning have been extensively studied in relation to grip preparation and the end-state-comfort effect (ESC) (Rosenbaum et al., 2012). Adult-like motor planning of ESC has been shown in several studies around age 10 (Scharoun Benson et al., 2018; Thibaut & Toussaint, 2010). In terms of more detailed measures of sequential actions, changes in motor planning kinematics have been described in toddler and preschool years (Chen et al., 2010; Claxton et al., 2003) with further refinement continuing at least into early school years (Domellöf et al., 2020). A transition period from more feedback-based exploratory strategies into a destabilization period followed by a more adult-like stable control of both feedforward and feedback strategies has been proposed to take place during early school-age (ages 7–10) (Jongbloed-Pereboom et al., 2016; Serrien & O’Regan, 2021; Thibaut & Toussaint, 2010). Furthermore, it has been proposed that this motor planning reorganization may be related to changes in multisensory integration in sensorimotor processes (Jongbloed-Pereboom et al., 2016; Serrien & O’Regan, 2021; Thibaut & Toussaint, 2010). However, there is a lack of detailed characterizations of typical motor planning development during early school-age, and little is known about how motor planning processes develop in children with ASD.

This longitudinal study used kinematic analysis to investigate the development of motor planning and performance of goal-directed sequential manual movements in a group of children with ASD comparing them with age-matched TD peers over early school-age. We manipulated the availability of initial visual information and precision affordances of the end-goal to examine different aspects of motor planning.

Two hypotheses were proposed for this study. The first hypothesis (1a–b) is related to the development of motor planning, while the second hypothesis (2) specifically focuses on the potential benefits of early available visual information for motor planning:

Hypothesis 1a: Both groups of children are expected to demonstrate improving efficiency in motor planning and performance as they age. However, it is anticipated that the ASD-group will not reach the same level of proficiency as the TD-group. This difference is expected to become more pronounced with older age and more challenging variations of the task.
Hypothesis 1b: It is also expected that the ASD-group will not demonstrate the same level of sequential movement chaining as the TD-group, particularly as they grow older.
Hypothesis 2: The benefits of having visual information available at an early stage for motor planning kinematics are expected to increase with age. However, it is anticipated that children with ASD will exhibit atypical benefits compared with their TD peers.

## Method

### Participants

Fourteen children with ASD (five girls; mean age: 7 years 9 months; range = 7y3m–8y8m) and 17 TD children (nine girls; mean age: 7 years 7 months; range = 6y11m–8y8m) initially participated in the study (age-level 1, A1). The children were re-invited for follow-up testing twice (A2, A3) with 1-year intervals (age range: ASD_A2_ = 8y4m–9y7m; ASD_A3_ = 9y3m–10y7m; TD_A2_ = 7y11m–9y8m; TD_A3_ = 8y11m–10y9m). In a study using a similar task, large consistent age effects were reported with groups of eight participants (Domellöf et al., 2020). Two boys with ASD declined participation after A1. Data from two behavioral assessments (caregiver reports) for these boys are also missing in the background participant information (Table 1).

**Table 1.** Descriptive characteristics of the children at first assessment (A1).

	ASD (*n* = 14)			TD (*n* = 17)		
	*M*	*SD*	Range	*M*	*SD*	Range
FSIQ	82.4	15.0	54–110	106.1	9.4	93–126
FRI	92.9	15.3	72–126	101.9	9.6	88–118
WMI	82.9	16.0	65–115	102.5	10.5	88–122
GEF	81.8^a^	26.0	21–99	25.5	22.0	1–71
MABC-2	23.5^a^	13.5	2–46^b^	4.4	4.4	0–14^b^
EHQ	0.72	0.25	0.25–1	0.93	0.12	0.56–1

FSIQ, Full-Scale Intelligence Quotient; FRI, Fluid Reasoning Index; WMI, Working Memory Index (FSIQ, FRI, WMI assessed by Wechsler Intelligence Scale for Children–Fifth Edition; WISC-V); GEF, %-rank of global executive composite score assessed by the Behavior Rating Inventory of Executive Function (BRIEF; caregiver ratings); MABC-2, total score on Movement Assessment Battery for Children–2 checklist (caregiver ratings); EHQ, absolute values of laterality index assessed by Edinburgh Handedness Questionnaire (caregiver ratings). ^a^ *n* = 12. ^b^ Scores indicating high likelihood of movement difficulties, *n*_ASD_ = 9, *n*_TD_ = 0. Significant group differences were evident for all measures, Mann–Whitney *U* Test (*p* < 0.05).

The children with ASD were identified through patient records at a local habilitation center. They had a formal ASD diagnosis established by the healthcare system and, at the time of invitation, had not been diagnosed with an intellectual disability. The TD children, recruited by advertisement at local schools and convenience sampling, had no established neurodevelopmental disorder diagnosis as an inclusion criterion. See Table 1 for additional participant information at first assessment (A1). All participants and caregivers provided their informed consent. The study received approval from the Umeå Regional Ethical Board registration (nr 2016/365-31).

### Community involvement

While parents or community providers were not directly involved in the research design or interpretation of results, the study protocol and implementation were informed by clinicians working with and parents of children with ASD.

### Sequential manual movement task

**Figure 1.**
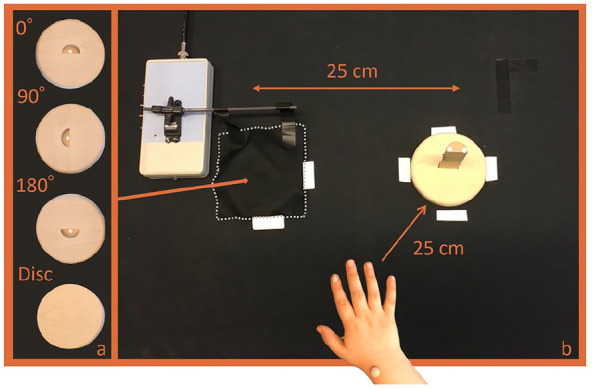
Panel (a) shows the four different presentations of the goal, and panel (b) illustrates the set-up of the occluded condition for a right-handed child, including marker placement. The semi-circular peg (straight side = 2.5 cm) is positioned in the start-holder to the right and the goal-holder is occluded under the cloth to the left, about to become visible.

The children performed different sequential manual tasks consisting of grasping a semi-circular peg from a start-holder and fitting it into a goal-slot centered on a goal-holder or placing it on a flat disk (goal-holder turned up-side down). The goal-slot was presented at three different orientations (0°, 90°, and 180° rotation relative to the peg orientation at start) (Figure 1). In half of the trials, the goal was fully visible prior to the commencement of the measurement (visual condition). In remaining trials, it was occluded with a cloth prior to measurement onset (occluded condition) and mechanically removed in synchronization with the onset. The children were asked to start when a beep-signal, synchronized with the measurement onset, sounded (visual condition) or when they saw the goal (occluded condition). The preferred hand was used for task performance with reversed set-up for left-handed compared with right-handed children (TD: all right-handed; ASD: five left-handed). Hand preference was assessed by caregiver ratings on an age-modified Edinburgh Handedness Questionnaire (Oldfield, 1971). Preference for the highly lateralized tasks writing/drawing was used if ratings showed no established hand preference (Fagard et al., 2015). The children were wearing Tobii eye tracking glasses to collect accompanying gaze behavior (outside the scope of this article).

Prior to the experiment, all children practiced the four tasks in the visual condition and the disk task in the occluded condition. If the child showed difficulties understanding/performing the task, it was practiced until it was understood. The experiment consisted of four blocks including all goal presentations in visual and occluded condition in a randomized order. At the end of each block, unsuccessful trials (e.g. bimanually assisting performance, clearly repetitive movements) were repeated (TD: n_A1_ = 10, n_A2_ = 7, n_A3_ = 3; ASD: n_A1_ = 50, n_A2_ = 17, n_A3_ = 17).

### Data acquisition

Movement recordings were performed by a five-camera optoelectronic system with a sampling frequency of 100 Hz (Oqus; Qualisys Inc.). To facilitate data preprocessing, each trial was concurrently video recorded. Passive reflective markers were attached to the wrist (radial styloid) (12 mm diameter) and the index finger (7 mm) of the performing hand. Two flat circular markers (5 mm) were attached with a 20 mm centroid distance on the peg top surface.

### Data processing

The Qualisys Track Manager software was used to combine information into three-dimensional (3D) data. Automatically filled data gaps (⩽10 frames) were inspected. Larger gaps were manually filled when feasible, using software interpolation methods facilitated by visual inspection of spatial/velocity profiles and marker projection on video recordings, and utilized subsequent inter-judge agreement.

**Figure 2.**
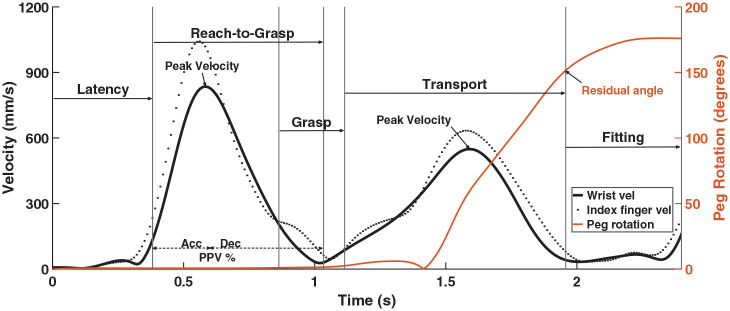
Illustration of the sequential movement subphases and extracted kinematic outcome variables, made by a 7-year-old TD child in the 180° goal orientation (visible condition). Left y-axis shows wrist and index finger velocity, and the right y-axis shows peg rotation angle.

The task performance was divided into five subphases: (1) latency, (2) reach-to-grasp (RTG), (3) grasp, (4) transport, and (5) fitting/placing (Figure 2; see Supplementary Table S1 for division criteria). In the data processing, 45 of the 2831 trials were excluded from further processing due to incorrect peg rotation during transport (indicating that the action was planned for an incorrect orientation) (TD: n_A1_ = 9, n_A2_ = 3, n_A3_ = 3; ASD: n_A1_ = 13, n_A2_ = 10, n_A3_ = 7). Parameter data in 96 trials were also excluded from further analysis due to missing data or false start (<100 ms).

### Outcome variables

Prior to extracting the kinematic parameters, data were smoothed with a forward and reversed 6 Hz Butterworth filter to a resulting order of 4 using in-house MATLAB (The Mathworks Inc.) scripts. Based on previous literature, extracted motor planning outcomes were latency, part peak velocity (PPV; the percentage of time when peak velocity occurs) of the wrist in RTG, peak velocity (PV) of the wrist in the RTG and transport phase, grip duration, and residual angle (RA; defined as the remaining percentage of the peg rotation angle needed at transport offset in the 180° and 90° orientations). Fitting duration served as the main outcome related to motor execution.

### Statistical analyses

A series of mixed linear models were performed to analyze the kinematic data (SPSS, version 28). All follow-up comparisons were made using Sidak correction. The duration outcomes (latency, grip duration and fitting duration; Ln(x)) and RA (Ln(x + 1)) were logarithmically transformed to improve normality. Significant (*p* < 0.05) outcomes are reported in the “Results” section. All main model results and the developmental patterns of change for each child group are presented in Supplementary Tables 2–4.

To investigate motor planning and performance development in the respective visual and occluded condition, the analyses for Hypothesis 1a and b were conducted in each condition separately. For each participant, all outcome variables were averaged for each orientation. Designated linear mixed effects models were then used to analyze differences. For Hypothesis 1a, age-level (A1–A3; repeated), group (TD/ASD), and orientation (four levels; two levels for RA) were included in the model and used as fixed main effects, with a random effect intercept for participants. Interactions between Age-Level × Group and Group × Orientation were specified. A main effect of age-level was expected as a sign of increasing efficiency in motor planning and performance in both child groups. It was expected that increasing motor planning efficiency would be shown as reduced latency in the visual condition, increased PPV, shorter grip duration, and a lower RA over age. No clear predictions could be made based on previous literature regarding the direction of change for PV in absolute terms. However, it was expected that motor performance efficiency would increase in terms of shorter fitting durations as individuals age. Accordingly, the hypothesized divergence in ASD development would be expressed as an interaction effect between age-level and group indicating atypical motor planning. The motor planning differences in the ASD group were anticipated to be more pronounced in more challenging task orientations.

To explore developmental patterns of chaining according to Hypothesis 1b, sensitive outcome measures related to chaining (PPV-RTG, PV-RTG, and PV-Transport) were investigated. Here, chaining is defined as initial movement modulation toward the end-goal difficulty of the task (lower PPV-RTG, PV-RTG, and PV-Transport for more difficult tasks). For Hypothesis 1b, age-level (A1–A3; repeated), group (TD/ASD), and orientation (four levels) were included in the model and used as fixed main effects, with a random effect intercept for participants. A three-way interaction between Age-Level × Group × Orientation was specified. The orientation effects within interactions were of main interest, where chaining would be expressed as evident orientation effects in respective groups at each age-level. In support of Hypothesis 1b, we predicted the ASD-group to display atypical chaining of sequential movements compared with the TD-group as expressed by a lack of statistically significant orientation effects in these analyses.

Hypothesis 2 was formulated to investigate changes in how initial visual information about the specific end-goal was integrated and used in motor planning. To test Hypothesis 2, the difference between the visual and occluded means, pooled across orientations, was calculated for each participant for all outcome variables separately. Designated linear mixed effects models were then used to analyze the derived variables. Age-level (A1–A3; repeated) and group (TD/ASD) were included in the model and used as fixed main effects, with a random effect intercept for participants. Interactions between Age-Level × Group were specified. A main effect of age-level was taken as an indication of developmental change in how much the children benefited from early available visual information. That is, seeing the end-goal in advance, prior to measurement onset, was expected to benefit motor planning. Increased latency in the occluded condition was expected with age (larger differences between conditions). Larger differences between conditions over age for PPV, grip duration, and RA, indicating increased motor planning benefits in the visual condition, were further proposed. Increasing condition differences for PV were not expected as increased motor planning in the visual condition would be better indicated by more PV differentiation between orientations (increased chaining) rather than change in absolute terms. Nor was increased benefit from the visual condition expected for fitting duration performance. This, since condition effects were predicted to be accounted for during the initial parts of the sequential movement as online corrections were possible. Regarding group differences, we expected a main effect of group or an Age-Level × Group interaction, indicating that the ASD-group would show limited or atypical benefit from early available visual information compared with the TD-group.

## Results

**Figure 3.**
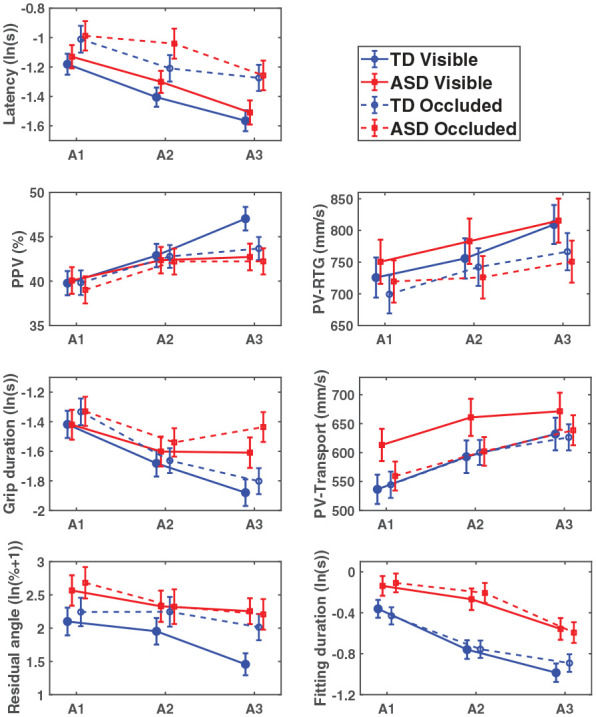
The graphs illustrate estimated mean values ± *SE* from the interaction between age-level and group over time, shown separately for each group in both the visual (solid line) and occluded (dotted line) condition.

**Figure 4.**
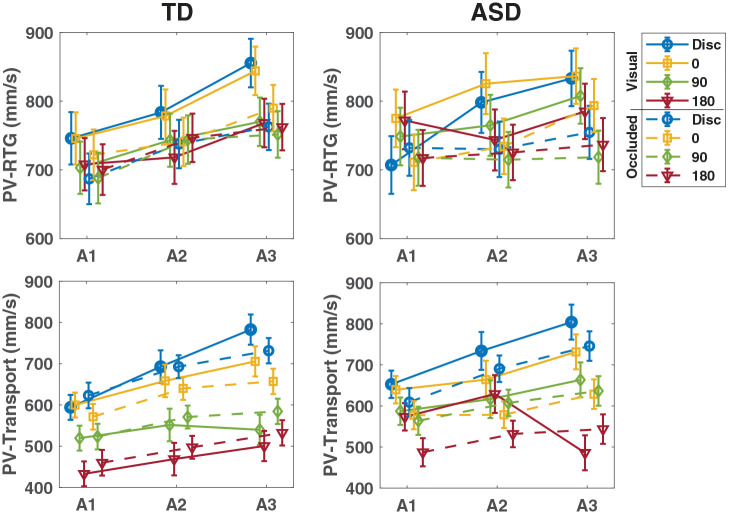
The graphs illustrate estimated mean values ± *SE* from the interaction between age-level and orientation over time for each group in both the visual (solid line) and occluded (dotted line) conditions. Both groups show lower peak velocities for more difficult orientations, more clearly differentiated in the TD- than ASD-group, particularly in the visual condition.

Table 2 presents descriptive (un-modeled) statistics of kinematic variables. The effects of age-level within respective group and condition for all outcomes are illustrated in Figure 3. Figure 4 illustrates the effects of orientation on peak velocity (PV) over time within respective group and condition.

**Table 2.** Descriptive statistics of kinematic variables for each condition (visual, occluded) divided by group (TD, ASD) over included age-levels (A1, A2, A3) and orientations (disk, 0°, 90°, 180°), respectively.

Kinematic variables	Group	*M* (*SD*) *Transformed mean value (SD)*						
		A1	A2	A3	Disk	0°	90°	180°
**Visual condition**								
Latency (s)	TD	0.33 (0.15) *–1.18 (0.39)*	0.25 (0.06) *–1.40 (0.24)*	0.22 (0.09) *–1.57 (0.34)*	0.26 (0.11) *–1.40 (0.34)*	0.28 (0.12) *–1.35 (0.38)*	0.26 (0.11) *–1.41 (0.34)*	0.28 (0.14) *–1.37 (0.40)*
	ASD	0.38 (0.30) *–1.13 (0.53)*	0.29 (0.13) *–1.30 (0.39)*	0.25 (0.15) *–1.51 (0.46)*	0.30 (0.22) *–1.32 (0.46)*	0.31 (0.22) *–1.31 (0.51)*	0.34 (0.23) *–1.25 (0.53)*	0.30 (0.21) *–1.34 (0.48)*
PPV-RTG (%)	TD	39.77 (4.89)	42.89 (5.40)	47.04 (6.40)	43.78 (6.80)	43.57 (5.95)	42.64 (5.84)	42.95 (6.79)
	ASD	40.07 (7.80)	42.08 (7.58)	42.46 (7.82)	43.01 (8.27)	41.19 (8.24)	41.36 (8.03)	40.29 (6.43)
PV-RTG (mm/s)	TD	725.7 (140.0)	755.8 (125.6)	809.3 (178.9)	795.0 (176.2)	789.3 (153.0)	738.7 (136.6)	731.5 (136.9)
	ASD	750.6 (149.1)	792.2 (155.1)	824.4 (146.5)	781.4 (163.7)	816.4 (153.5)	777.6 (143.8)	772.8 (149.9)
Grip duration (s)	TD	0.27 (0.14) *–1.42 (0.46)*	0.20 (0.09) *–1.68 (0.43)*	0.17 (0.08) *–1.88 (0.42*)	0.20 (0.09) *–1.71 (0.49)*	0.21 (0.13) *–1.68 (0.48)*	0.20 (0.08) *–1.68 (0.38)*	0.24 (0.14) *–1.57 (0.53)*
	ASD	0.27 (0.12) *–1.42 (0.49)*	0.22 (0.13) *–1.62 (0.51)*	0.24 (0.21) *–1.63 (0.60)*	0.22 (0.11) *–1.62 (0.47)*	0.23 (0.14) *–1.59 (0.53)*	0.26 (0.18) *–1.54 (0.62)*	0.28 (0.19) *–1.44 (0.53)*
PV-Transport (mm/s)	TD	536.5 (127.5)	592.8 (140.6)	632.0 (178.5)	689.9 (169.0)	654.6 (114.5)	536.9 (100.4)	467.3 (113.6)
	ASD	613.1 (154.0)	672.3 (195.2)	682.7 (181.9)	732.9 (223.9)	683.7 (153.8)	627.9 (134.6)	571.0 (151.2)
Residual angle (%)	TD	14.35 (16.94) *2.10 (1.21)*	11.08 (11.42) *1.95 (1.15)*	5.19 (6.14) *1.46 (0.87)*	*–*	*–*	4.85 (6.06) *1.27 (1.01)*	15.57 (15.31) *2.40 (0.91)*
	ASD	21.39 (20.04) *2.57 (1.20)*	19.54 (20.85) *2.31 (1.38)*	15.45 (17.64) *2.24 (1.19)*	*–*	*–*	11.85 (15.32) *1.79 (1.31)*	26.01 (20.79) *2.97 (0.85)*
Fitting duration (s)	TD	0.83 (0.48) *–0.36 (0.63)*	0.60 (0.35) *–0.76 (0.86)*	0.49 (0.29) *–0.99 (0.88)*	0.27 (0.25) *–1.71 (0.94)*	0.61 (0.22) *–0.56 (0.33)*	0.71 (0.30) *–0.41 (0.38)*	0.97 (0.45) *–0.42 (0.45)*
	ASD	1.14 (0.73) *–0.14 (0.87)*	1.04 (0.74) *–0.27 (0.96)*	0.89 (0.95) *–0.56 (1.07)*	0.42 (0.41) *–1.42 (1.16)*	0.86 (0.36) *–0.24 (0.41)*	1.13 (0.57) *0.02 (0.46)*	1.71 (1.09) *0.26 (0.69)*
**Occluded condition**								
Latency (s)	TD	0.39 (0.17) *–1.01 (0.38)*	0.31 (0.09) *–1.21 (0.29)*	0.30 (0.11) *–1.27 (0.38)*	0.34 (0.15) *–1.15 (0.40)*	0.33 (0.14) *–1.17 (0.38)*	0.33 (0.12) *–1.17 (0.32)*	0.33 (0.14) *–1.17 (0.38)*
	ASD	0.46 (0.39) *–0.99 (0.60)*	0.40 (0.21) *–1.03 (0.48)*	0.32 (0.13) *–1.25 (0.46)*	0.41 (0.27) *–1.06 (0.56)*	0.38 (0.29) *–1.14 (0.54)*	0.41 (0.25) *–1.02 (0.49)*	0.39 (0.39) *–1.12 (0.55)*
PPV-RTG (%)	TD	39.82 (5.20)	42.78 (5.67)	43.68 (6.12)	42.46 (5.57)	42.81 (5.74)	42.14 (6.29)	40.97 (5.92)
	ASD	39.02 (7.94)	41.90 (7.87)	41.91 (7.11)	41.49 (7.58)	40.58 (8.93)	40.83 (7.20)	40.45 (7.41)
PV-RTG (mm/s)	TD	699.3 (135.1)	742.2 (111.8)	766.4 (176.2)	729.0 (139.5)	750.8 (145.4)	727.9 (149.5)	736.4 (150.6)
	ASD	719.4 (151.1)	729.1 (134.8)	753.7 (126.3)	740.0 (140.7)	746.3 (145.3)	719.2 (130.1)	728.0 (141.2)
Grip duration (s)	TD	0.30 (0.19) *–1.33 (0.48)*	0.20 (0.09) *–1.66 (0.38)*	0.18 (0.07) *–1.80 (0.36)*	0.20 (0.08) *–1.66 (0.35)*	0.22 (0.10) *–1.59 (0.39)*	0.21 (0.10) *–1.67 (0.44)*	0.28 (0.22) *–1.47 (0.58)*
	ASD	0.29 (0.12) *–1.33 (0.45)*	0.25 (0.13) *–1.54 (0.52)*	0.30 (0.28) *–1.43 (0.64)*	0.28 (0.24) *–1.46 (0.57)*	0.26 (0.13) *–1.46 (0.50)*	0.27 (0.17) *–1.44 (0.53)*	0.31 (0.21) *–1.35 (0.57)*
PV-Transport (mm/s)	TD	544.3 (137.3)	600.2 (129.2)	626.3 (153.3)	682.5 (138.4)	622.9 (117.1)	559.4 (121.9)	496.5 (128.5)
	ASD	559.4 (143.3)	604.7 (123.1)	641.3 (134.7)	678.5 (140.5)	596.7 (124.1)	602.8 (122.6)	520.4 (120.7)
Residual angle (%)	TD	14.95 (15.18) *2.24 (1.15)*	14.78 (16.10) *2.25 (1.13)*	13.75 (17.62) *2.02 (1.26)*	*–*	*–*	7.36 (8.37) *1.58 (1.13)*	21.63 (18.84) *2.76 (0.89)*
	ASD	26.50 (26.97) *2.68 (1.31)*	21.07 (25.41) *2.28 (1.46)*	13.88 (16.31) *2.16 (1.13)*	*–*	*–*	11.35 (14.19) *1.78 (1.30)*	30.25 (27.71) *2.99 (1.02)*
Fitting duration (s)	TD	0.79 (0.46) *–0.43 (0.69)*	0.58 (0.30) *–0.76 (0.75)*	0.52 (0.29) *–0.89 (0.83)*	0.25 (0.22) *–1.69 (0.80)*	0.61 (0.25) *–0.56 (0.35)*	0.72 (0.23) *–0.38 (0.32)*	0.94 (0.40) *–0.14 (0.37)*
	ASD	1.19 (0.82) *–0.11 (0.87)*	1.10 (0.88) *–0.21 (0.95)*	0.83 (0.67) *–0.59 (1.10)*	0.44 (0.44) *–1.42 (1.21)*	0.89 (0.33) *–0.18 (0.34)*	1.12 (0.52) *0.02 (0.42)*	1.75 (1.07) *0.40 (0.55)*

### Investigation of dynamic effects of development and task difficulty on motor planning and performance (Hypothesis 1a)

Hypothesis 1a was investigated separately in each condition (visual, occluded).

### Latency

#### Visual condition

A main effect of age-level (*F*(2, 118.3) = 31.89, *p* < 0.001) was found for latency. Latencies became shorter with increasing age (A1 > A2 > A3; *p* < 0.001).

#### Occluded condition

A main effect of age-level (*F*(2, 118.5) = 20.36, *p* < 0.001) was also found in the occluded condition where latencies became shorter with increasing age (A1 > A2 > A3; *p* ⩽ 0.013).

### PPV-RTG

#### Visual condition

A main effect of age-level (*F*(2, 118.1) = 26.90, *p* < 0.001) and a significant Age-Level × Group interaction (*F*(2, 118.1) = 7.78, *p* < 0.001) were found for PPV-RTG. Both groups had later PPV-RTG (indicating a shorter relative deceleration phase) with increasing age, evident between each year in the TD-group (A1 < A2 < A3; *p* < 0.001) but not between A2 and A3 in the ASD-group (A1 < A2 and A1 < A3; *p* ⩽ 0.046). The ASD-group further showed earlier PPV-RTG (a longer relative deceleration phase) compared with the TD-group at A3 (*p* = 0.038).

#### Occluded condition

A main effect of age-level (*F*(2, 117.5) = 13.31, *p* < 0.001) was found for PPV-RTG where the children showed later PPV-RTG with increasing age (A1 < A2 and A1 < A3; *p* < 0.001), but not statistically significant between A2 and A3.

### PV-RTG

#### Visual condition

Main effects of age-level (*F*(2, 118.2) = 14.82, *p* < 0.001) and orientation (*F*(3, 82.8) = 10.39, *p* < 0.001) were found for PV-RTG. The PV-RTG was increasing with increasing age (A1 < A3 and A2 < A3; *p* ⩽ 0.006), but not statistically significant between A1 and A2. Regarding the orientation effect, PV-RTGs were lower in the more difficult goal orientations compared with the less difficult ones (180° < 0°; 180° < disk; 90° < 0°; 90° < disk; *p* ⩽ 0.019).

#### Occluded condition

Similarly, main effects of age-level (*F*(2, 118.7) = 6.90, *p* < 0.001) and orientation (*F*(3, 82.6) = 4.14, *p* = 0.009) were found in the occluded condition. The PV-RTG was increasing with increasing age, however only statistically significant between the 2-year interval of A1 and A3 (A1 < A3; *p* < 0.001). Regarding the orientation effect, the PV-RTGs were lower in 90° compared with 0° goal orientation (*p* = 0.005).

### Grip duration

#### Visual condition

A main effect of age-level (*F*(2, 116.6) = 18.46, *p* < 0.001) and orientation (*F*(3, 83.8) = 4.76, *p* = 0.004) and a significant Age-Level × Group interaction (*F*(2, 116.6) = 3.66, *p* = 0.029) were found for grip duration. Both groups exhibited shorter grip durations with increasing age. This was apparent between each year in the TD-group (A1 > A2 > A3; *p* ⩽ 0.002) and between A1 and A2 (but not between A1 and A3) in the ASD-group (A1 > A2; *p* = 0.041). Independent of group, longer grip durations were shown in 180° compared with the 0° and the disk goal orientations (180° > 0°; 180° > disk; *p* ⩽ 0.030).

#### Occluded condition

A main effect of age-level (*F*(2, 119.3) = 15.82, *p* < 0.001) and orientation (*F*(3, 83.6) = 6.85, *p* < 0.001) and a significant Age-Level × Group interaction (*F*(2, 119.3) = 5.75, *p* = 0.004) were also found in the occluded condition. Both groups showed shorter grip durations with increasing age, significantly different between each year in the TD-group (A1 > A2 > A3; *p* ⩽ 0.045) and between A1 and A2 (but not between A1 and A3) in the ASD-group (A1 > A2; *p* = 0.022). The ASD-group further showed significantly longer grip durations compared with the TD-group at A3 (*p* = 0.009). Orientation effects described longer grip durations in the 180° goal orientation (180° > 90°; 180° > 0°; 180° > disk; *p* ⩽ 0.021).

### PV-transport

#### Visual condition

Main effects of age-level (*F*(2, 115.2) = 13.86, *p* < 0.001) and orientation (*F*(3, 86.8) = 43.02, *p* < 0.001) and a significant Group × Orientation interaction (*F*(3, 86.8) = 2.81, *p* = 0.044) were found on PV-Transport. The PV-Transport increased with increasing age, but not statistically different between A2 and A3 (A1 < A2 and A1 < A3; *p* ⩽ 0.002). Regarding the orientation effect, PV-Transports were lower in the more difficult goal orientations compared with the less difficult ones (180° < 90°; 180° < 0°; 180° < disk; 90° < 0°; 90° < disk; *p* ⩽ 0.003). This pattern was less differentiated in the ASD-group (180° < 0°; 180° < disk; 90° < disk; *p* ⩽ 0.003) than TD-group (180° < 90°; 180° < 0°; 180° < disk; 90° < 0°; 90° < disk; *p* ⩽ 0.003). The ASD-group also had higher PV-Transport than the TD-group in the 180° orientation (*p* = 0.013).

#### Occluded condition

Main effects of age-level (*F*(2, 117.4) = 17.08, *p* < 0.001) and orientation (*F*(3, 84.6) = 56.21, *p* < 0.001) and a significant Group × Orientation interaction (*F*(3, 84.6) = 2.84, *p* = 0.043) were also found in the occluded condition. The PV-Transport increased with increasing age (A1 > A2 > A3; *p* ⩽ 0.014). In the orientation effect, lower PV-Transports were evident for more difficult orientations (180° < 90°; 180° < 0°; 180° < disk; 90° < disk; 0° < disk; *p* < 0.001). The interaction showed within-group orientation effects; in the ASD-group (180° < 90°; 180° < 0°; 180° < disk; 90° < disk; 0° < disk; *p* ⩽ 0.004), in the TD-group (180° < 90°; 180° < 0°; 180° < disk; 90° < 0°; 90° < disk; 0° < disk; *p* ⩽ 0.008).

### Residual angle

#### Visual condition

Main effects of age-level (*F*(2, 59.4) = 4.56, *p* = 0.014), group (*F*(1, 31.1) = 6.41, *p* = 0.017), and orientation (*F*(1, 28.2) = 106.74, *p* < 0.001) were found for RA. RA became lower with increasing age, statistically different between the 2-year interval of A1 and A3 (A1 < A3; *p* = 0.014). The ASD-group showed higher RA compared with the TD-group, mainly attributable to significant group differences at A3 (*p* = 0.003). Furthermore, RA was lower for 90° compared with 180° orientation (*p* < 0.001).

#### Occluded condition

In the occluded condition, there was only a main effect of orientation (*F*(1, 29.3) = 70.40, *p* < 0.001) for RA where RA was lower for 90° compared with 180° orientation (*p* < 0.001). None of the groups displayed significantly lower RA with increasing age.

### Fitting duration

#### Visual condition

Main effects of age-level (*F*(2, 118.3) = 27.88, *p* < 0.001), group (*F*(1, 31.0) = 11.06, *p* = 0.002), and orientation (*F*(3, 88.5) = 109.02, *p* < 0.001) were found for fitting duration. Fitting durations decreased as age increased (A1 > A2 > A3; *p* < 0.001). The ASD-group showed longer fitting durations compared with the TD-group. Although no interaction effects were found, understanding of the results may be helped by evident group differences at A2 (*p* < 0.001) and A3 (*p* = 0.003) and in the 180° (*p* = 0.003) and the 90° (*p* = 0.007) orientation. The orientation effect was characterized by longer fitting durations for more difficult orientations (180° > 90°; 180° > 0° 180° > disk; 90° > disk; 0° > disk; *p* ⩽ 0.016).

#### Occluded condition

In the occluded condition, main effects of age-level (*F*(2, 118.1) = 23.58, *p* < 0.001), group (*F*(1, 30.5) = 12.93, *p* = 0.001), and orientation (*F*(3, 88.0) = 123.47, *p* < 0.001) were found, and a significant Age-Level × Group interaction (*F*(2, 118.1) = 3.62, *p* = 0.030). Both groups had shorter fitting durations with increasing age, but not statistically different between A2 and A3 in the TD-group (A1 > A2 and A1 > A3; p < .001) and between A1 and A2 in the ASD-group (A1 > A3 and A2 > A3; *p* < 0.001). The ASD-group displayed longer fitting durations compared with the TD-group, evident at all age-levels (*p*_A1_ = 0.013; *p*_A2_ < 0.001; *p*_A3_ = 0.028). Again, although no interaction effect was found, understanding of these results may be aided by evident group differences in the 180° (*p* = 0.001), 90° (*p* = 0.010) and 0° (*p* = 0.015) orientation. The orientation effect was characterized by longer fitting durations for more difficult orientations (180° > 90°; 180° > 0°; 180° > disk, 90° > disk; 0° > disk; *p* ⩽ 0.009).

### Investigating chaining of the movement over age (Hypothesis 1b)

Hypothesis 1b was examined separately in each condition (visual, occluded), specifically focusing on the orientation effects within the three-way interactions, indicating chaining.

#### PPV-RTG

For both the TD- and ASD-group, no evident orientation effects were shown at any age-level in neither the visual nor occluded condition.

#### PV-RTG

In the visual condition, although not reflected by a significant three-way interaction effect (*F*(17, 102.6) = 0.95, *p* *=* 0.518), a significant orientation effect was observed in the TD-group at A3 (*p* = 0.003) with lower PV-RTGs for the more difficult orientations (180° < disk; 90° < disk; *p* ⩽ 0.028). In the occluded condition, there were no evident orientation effects.

#### PV-transport

In the visual condition, a significant three-way interaction effect (*F*(17, 100.2) = 3.08, *p* < 0.001) was revealed. Significant orientation effects were shown at all age-levels in the TD-group (*p*_A1_ < 0.001; *p*_A2_ < 0.001; *p*_A3_ < 0.001) and at A1 and A3 in the ASD-group (*p*_A1_ = 0.026; *p*_A3_ < 0.001). PV-Transports were lower for more difficult orientations, however less differentiated in the ASD-group (ASD_A1_: nonsignificant; ASD_A3_:180° < 90°; 180° < 0°; 180° < disk; 90° < disk; *p* ⩽ 0.026) than in the TD-group (TD_A1_:180° < 90°; 180° < 0°; 180° < disk; 90° < 0°; 90° < disk; TD_A2_:180° < 0°; 180° < disk; 90° < disk; TD_A3_:180° < 0°; 180° < disk; 90° < 0°; 90° < disk; *p* ⩽ 0.047).

In the occluded condition, no significant three-way interaction effect (*F*(17, 121.6) = 0.82, *p* = 0.663) was evident as orientation effects were shown at all age-levels in both groups (TD: *p*_A1_ < 0.001; *p*_A2_ < 0.001; *p*_A3_ < 0.001) (ASD: *p*_A1_ = 0.016; *p*_A2_ < 0.001; *p*_A3_ < 0.001). Lower PV-Transports for the more difficult orientations were shown in both the ASD-group (ASD_A1_: 180° < disk; ASD_A2_: 180° < disk; 0° < disk; ASD_A3_: 180° < disk; 90° < disk; 0° < disk; *p* ⩽ 0.047) and the TD-group (TD_A1_: 180° < 0°; 180° < disk; 90° < disk; TD_A2_: 180° < 0°; 180° < disk; 90° < disk; TD_A3_: 180° < 0°;180° < disk; 90° < disk; *p* ⩽ 0.031).

### Investigating changes related to early available visual information as beneficial for motor planning and performance (Hypothesis 2)

#### Latency difference between conditions

No significant age-level or group effects were found, indicating that there was no evident increase in latency condition difference over age and no difference between groups.

#### PPV-RTG difference between conditions

A main effect of age-level (*F*(2, 28.2) = 6.26, *p* = 0.006) and a significant Age-Level × Group interaction (*F*(2, 28.2) = 5.90, *p* = 0.007) were found on the PPV-RTG condition difference. Follow-up analyses of the interaction effect showed that the TD-group had larger PPV-RTG condition difference (higher PPV-RTG in visual than occluded condition) with increasing age. This was significant between A2 and A3 (A1 < A3 and A2 < A3; *p* ⩽ 0.005). In addition, at A3, the ASD-group showed smaller PPV-RTG condition difference (i.e. expressed reduced benefit from seeing the end-goal in advance for PPV-RTG) compared with the TD-group (*p* = 0.026).

#### PV-RTG difference between conditions

A main effect of group (*F*(1, 27.3) = 5.06, *p* = 0.033) was found on PV-RTG condition difference. The ASD-group showed larger PV-RTG condition difference (higher PV-RTG in the visual than the occluded condition) compared with the TD-group.

#### Grip duration difference between conditions

A main effect of group (*F*(1, 26.7) = 4.61, *p* = 0.041) was found on the grip duration condition difference. The ASD-group showed larger grip duration condition difference (shorter grip duration in the visual than occluded condition) compared with the TD-group.

#### PV-Transport difference between conditions

A main effect of group (*F*(1, 28.2) = 7.90, *p* = 0.009) was found on PV-Transport condition difference. The ASD-group showed larger PV-Transport condition difference (higher PV-Transport in the visual than occluded condition) compared with the TD-group.

#### RA difference between conditions

A significant Age-Level × Group interaction (*F*(2, 28.1) = 3.99, *p* = 0.030) was found on the RA condition difference. Follow-up analysis showed that the TD-group had increased RA condition difference (lower RA in the visual than occluded condition) compared with the ASD-group at A3 (*p* = 0.030).

#### Fitting duration difference between conditions

No significant effects of age-level or group were found, indicating that there was no increasing benefit from seeing the end-goal in advance in terms of fitting duration.

## Discussion

In support of the study hypotheses, both the TD- and ASD-group showed increasing efficiency in motor planning and performance over early school-age in all but one outcome measure. The exception being the RA in the occluded condition. The children also displayed increased chaining of the movement sequences over age, although atypically expressed in the visual condition in the ASD-group. In general, developmental change in the ASD-group appeared to be atypical in several outcomes in the visual condition with more pronounced differences arising at older age.

### Motor planning development and relations to visuomotor integration

The observed atypical motor planning in the visual condition in the ASD-group could be related to difficulties with integrating early available visual information in the initial motor plan. The reliance on visual information in motor planning appeared atypical over time in the ASD-group. Seeing the end-goal in advance of measurement onset did not seem to facilitate motor planning development to the same extent as for the TD-group. This interpretation is supported by findings in the visual condition of greater dependence on feedback control processes in the prehension movement (lower PPV), less predictive coordination of translations and rotations in the rotation of the peg (higher RA) and atypical PV adjustments toward task difficulty in the ASD compared with the TD-group, most evident at older age. These developmental trajectory differences did not emerge in the occluded condition, potentially due to the persistent use of more exploratory strategies by both groups in this condition (indicated by the short latencies observed at all ages).

Over the 3 years, developmental progression was observed for PPV-RTG in the visual condition in the TD-group, while there was no difference between A2 and A3 in the ASD-group. Thus, at A3, the ASD-group was characterized by less reliance on feedforward planning in the reaching phase (lower PPV). In the occluded condition, PPV-RTG change was only present between A1 and A2 for both child groups, and no group difference was found.

Similarly, the increasingly predictive rotation of the peg (lower RA) over age was also principally related to the visual condition. No change with age appeared in the occluded condition. The ASD-group showed increased RA compared with the TD-group mainly attributable to differences at A3. The less predictive object adjustments seen in the ASD-group are in line with previous research findings (Bäckström et al., 2021).

Furthermore, atypical PV was seen in the visual condition in the ASD-group. It is well known that movement velocity is affected by end-point difficulty in neurotypical adults, where lower velocity for more difficult goals can be seen (Wong et al., 2015). When it comes to the development of sequential motor planning in early school-age children, it is difficult to know what to expect in relation to peak velocity. Peak velocities have been reported to increase, decrease, or remain unchanged with age across various tasks (Kuhtz-Buschbeck et al., 1998; Olivier & Bard, 2000; Olivier et al., 2007). In this study, both the TD- and the ASD-group generally showed larger PV with increasing age. A group difference was, however, found for the most difficult 180° orientation in the visual condition transport phase, where the ASD-group exhibited higher PV-Transport than the TD-group. This finding indicates a reduction in anticipatory velocity adjustment (indicative of less chaining) in the most difficult task. Faster movements in early school-age children with ASD have been shown (Mari et al., 2003; Rodgers et al., 2019) and have been related to less adjustment relative to task difficulty (Rodgers et al., 2019).

Latency decreased over age in both visual and occluded condition for the children overall, and no group differences were present. Longer latencies compared with TD are commonly observed in older individuals with ASD (Sacrey et al., 2014), probably serving as a compensatory function for less optimal motor planning (Rodgers et al., 2019). The lack of group differences for latency in this study is, however, in line with previously reported results in early school-age children with ASD (Bäckström et al., 2021; Dowd et al., 2012; Rodgers et al., 2019). Inconsistencies in findings between studies may relate to differences in the ages studied. At younger ages, a possible explanation for the absence of group differences in latency may stem from generally immature inhibition mechanisms (Domellöf & Säfström, 2020) or an insufficient capacity to plan the substeps of sequential movements. Support of the latter suggestion is found in the decrease in grip duration with age in the TD-group in our study. In the ASD-group however, grip duration decreased between A1 and A2 but then increased, rendering grip duration at A3 not significantly different from A1 or A2. The ASD-group also showed prolonged grip duration compared with the TD-group in the occluded condition at A3. These findings could indicate a budding compensatory strategy developing in the ASD-group, where the grip phase functionally works as a “second latency” to update the upcoming movement plan.

Taken together, the transition from more exploratory strategies to more adult-like feedforward strategies, observed in typical motor planning development at this age (Jongbloed-Pereboom et al., 2016; Serrien & O’Regan, 2021; Thibaut & Toussaint, 2010), seems atypical in ASD, but almost exclusively in the presence of early available visual information.

Although motor planning seemed atypical in the ASD-group mainly in the visual condition, group differences in fitting duration were evident in both conditions. Longer fitting durations were mainly attributed to the orientations requiring peg rotation. However, it is possible that the occluded condition might have been more challenging than the visual condition for the ASD-group in fitting. This suggestion is indicated by decreases in fitting durations at an earlier age in the TD-group than the ASD-group in the occluded condition. Furthermore, group differences in the occluded condition were observed in all orientations except the easiest (disk) and across all age-levels. On the contrary, differences in the visual condition were mainly attributed to performance at A2 and A3 as well as to the two most difficult orientations (180° and 90°). These findings are expected as previous research has shown that children with ASD exhibit slower movements on tasks that are more challenging (Yang et al., 2014).

### Chaining of sequential movements

With regard to chaining, it was mainly in the visual condition that development in the ASD-group appeared to be atypical. The TD-group seemed to be aided by early available visual information in the process of integrating sequential movements. Indications of chaining from the PV-RTG were observed in the TD-group at A3 in the visual condition exclusively. In the ASD-group, no indications of chaining in PV-RTG were noticeable in neither the visual nor occluded condition. Signs of adjustments of reach-to-grasp velocity in relation to end-task difficulty in sequential movements have been shown as early as for preschoolers (Claxton et al., 2003) although not consistently (Wilmut et al., 2013). Conflicting findings may relate to the effort needed to adjust the prehension movement during the first motor act and how rewarding this will be for the onward movement (Wilmut et al., 2013). Compared with earlier studies, the present task has a more complex grasp component, probably affecting overall PV-RTG adjustments. This might also explain the lack of orientation effects for PPV-RTG.

For PV-Transport, orientation effects could be observed in both conditions already at A1 in the TD-group. Anticipatory velocity adjustment then became increasingly differentiated with age. For the ASD-group, the pattern was less clear. Goal-directed adjustments of PV-Transport were noticeable already at A1 in the occluded condition, and then continuously present at later ages. This stands in contrast to indications of chaining being observed first at A3 in the visual condition. One explanation for these more unexpected results may be found in relation to initial goal visibility. Rather than facilitating, it appeared as early available visual information distracted global processing in the group of children with ASD. These findings indicate atypical development of sequential movement planning in ASD and add support to previous findings of chaining difficulties in children with ASD (Bäckström et al., 2021; Cavallo et al., 2018; Fabbri-Destro et al., 2009; Forti et al., 2011). It has been shown that low-level perception processes with a strong preference for the local over the global is evident in sensory processing in adults with ASD (Robertson & Baron-Cohen, 2017). Speculatively, this could be related to findings of atypical connectivity between medial prefrontal cortex and other brain regions in ASD (Leisman et al., 2023). Thus, the issues with sequencing displayed by the developing children with ASD observed here might partly relate to atypical neural connectivity becoming increasingly evident with age.

### Developmental changes in the benefits of early available visual information on motor planning

Although latency decreased over age in both visual and occluded conditions, neither child group seemed to show an increasing latency condition difference with age. The rudimentary task-goal of placing the peg seems sufficient to initiate movement in this kind of task which affords online corrections. These results are in line with previous findings of shorter sequential movement latencies in both 6- and 10-year-old TD children compared with adults (Domellöf et al., 2020). However, increasing benefits of early available visual information of the specific end-goal (visual condition) were seen for PPV-RTG and RA. This indicates implicit age-level gains for feedforward planning. For PPV-RTG, only the TD-group showed increased benefits with age of seeing the end-goal in advance of measurement onset, evident between A2 and A3. The different developmental trajectories resulted in group differences at A3, where the ASD-group showed less benefits of early available visual information on reach-to-grasp feedforward processes compared with the TD-group. Similar results were shown for RA where the ASD-group benefited less than the TD-group of seeing the end-goal in advance at A3. Significant group effects were further shown on PV condition differences in terms of larger PV condition difference (higher peak velocities in the visual condition) for the ASD-group compared with the TD-group in both PV-RTG and PV-Transport. Thus, rather than adjusting toward end-goal difficulty, children with ASD may have generated a general plan for an overall easier target in the visual condition. This interpretation is also supported by the previously discussed lack of PV adjustment toward more difficult tasks for the ASD-group in the visual condition.

Taken together, the results related to Hypothesis 2 indicate that the TD children seem to benefit from early available visual information, resulting in improved motor planning. This improvement became more apparent as they got older and relied more on visual cues for action prediction. These results strengthen previous suggestions of reorganization of visuomotor integration at these ages, affecting typical motor planning development (Jongbloed-Pereboom et al., 2016; Serrien & O’Regan, 2021; Thibaut & Toussaint, 2010). However, children with ASD did not seem to show the same advantage in action prediction from early available visual information. Results are in line with previous findings of difficulties to consider all available visual information to modulate movement in children with ASD (Dowd et al., 2012). As such, this supports proposals of atypical visuomotor integration in ASD (Dowd et al., 2012; Lidstone & Mostofsky, 2021), in turn affecting motor planning development.

As pointed out by other studies (Kuhtz-Buschbeck et al., 1999; Rodgers et al., 2019), the dynamic interactions between age and task affect the generalization of behavioral data beyond similar tasks and age groups. This study highlights the importance of considering task, age, and the age range in future studies and to better explain inconsistencies between previous findings on motor planning in ASD. Longitudinal studies conducted at various ages are necessary to enhance our understanding of this neurodevelopmental disorder (Lord et al., 2020). In addition, this study does not provide information on the specificity of motor planning problems in individuals with ASD and how these problems are related to differences in cognitive abilities during development. Future studies should consider this important aspect, as cognitive challenges are commonly observed in individuals with ASD (Lord et al., 2020). The generalizability of our findings may be impacted by the relatively small sample sizes and the neurodiversity commonly observed in children with ASD (Table 1). Hence, continued longitudinal research is necessary to validate our findings and explore how motor planning processes develop in children with ASD. Furthermore, previous findings suggest that embodied motor representation may contribute to the formation of representations of others (Cook, 2016). Therefore, our findings can inform not only future motor interventions but possibly also interventions targeting social skills.

## Conclusion

In this study, we observed an increased efficiency in motor planning and performance of sequential manual movements with age in both children with ASD and TD children. Development in the ASD-group was, however, atypical in several respects, with group differences emerging at older age. The longitudinal findings from this study support the idea that increased use of visual information in action prediction promotes motor planning development in early school-age TD children, which aligns with suggestions from previous cross-sectional studies. In contrast, motor planning development in ASD did not appear to be facilitated to the same extent by increased reliance on visual information. Our longitudinal findings suggest that divergent motor planning development in ASD at an early school-age is associated with atypical visuomotor integration and global processing of sensory information.

## Supplemental Material

sj-docx-1-aut-10.1177_13623613241311333 – Supplemental material for Atypical development of sequential manual motor planning and visuomotor integration in children with autism at early school-age: A longitudinal kinematic study Supplemental material, sj-docx-1-aut-10.1177_13623613241311333 for Atypical development of sequential manual motor planning and visuomotor integration in children with autism at early school-age: A longitudinal kinematic study by Anna Bäckström, Anna-Maria Johansson, Thomas Rudolfsson, Louise Rönnqvist, Claes von Hofsten, Kerstin Rosander and Erik Domellöf in Autism

sj-docx-2-aut-10.1177_13623613241311333 – Supplemental material for Atypical development of sequential manual motor planning and visuomotor integration in children with autism at early school-age: A longitudinal kinematic study Supplemental material, sj-docx-2-aut-10.1177_13623613241311333 for Atypical development of sequential manual motor planning and visuomotor integration in children with autism at early school-age: A longitudinal kinematic study by Anna Bäckström, Anna-Maria Johansson, Thomas Rudolfsson, Louise Rönnqvist, Claes von Hofsten, Kerstin Rosander and Erik Domellöf in Autism

sj-docx-3-aut-10.1177_13623613241311333 – Supplemental material for Atypical development of sequential manual motor planning and visuomotor integration in children with autism at early school-age: A longitudinal kinematic study Supplemental material, sj-docx-3-aut-10.1177_13623613241311333 for Atypical development of sequential manual motor planning and visuomotor integration in children with autism at early school-age: A longitudinal kinematic study by Anna Bäckström, Anna-Maria Johansson, Thomas Rudolfsson, Louise Rönnqvist, Claes von Hofsten, Kerstin Rosander and Erik Domellöf in Autism

sj-docx-4-aut-10.1177_13623613241311333 – Supplemental material for Atypical development of sequential manual motor planning and visuomotor integration in children with autism at early school-age: A longitudinal kinematic study Supplemental material, sj-docx-4-aut-10.1177_13623613241311333 for Atypical development of sequential manual motor planning and visuomotor integration in children with autism at early school-age: A longitudinal kinematic study by Anna Bäckström, Anna-Maria Johansson, Thomas Rudolfsson, Louise Rönnqvist, Claes von Hofsten, Kerstin Rosander and Erik Domellöf in Autism
