# Supplementary material for: Atypical development of sequential manual motor planning and visuomotor integration in children with autism at early school-age: A longitudinal kinematic study
Source: Autism. 2025 Jan 6;29(6):1510–23. doi: 10.1177/13623613241311333 (PMC12089664; doi:10.1177/13623613241311333)
Supplement: sj-docx-1-aut-10.1177_13623613241311333 – Supplemental material for Atypical development of sequential manual motor planning and visuomotor integration in children with autism at early school-age: A longitudinal kinematic study [file sj-docx-1-aut-10.1177_13623613241311333.docx]

| **Supplementary Table S1**  *Criteria for the division of the sequential movement into the five sub-phases (applied on unfiltered data).* | | |
| --- | --- | --- |
| **Sub-phase** | **Onset definition** | **Offset definition** |
| Latency | Start of measurement | When the wrist marker exceeded a velocity of 20 mm/s in the initiation of prehension |
| Reach-to-grasp (RTG) | Defined as latency offset | When a low point in velocity (≤10% of peak velocity) was reached by the wrist marker after first peg touch (prompt increase in peg marker velocity) |
| Grasp | Defined as first peg touch (prompt increase in peg marker velocity) | When achieving 1 mm upwards movement of the peg markers during lifting the peg out of the start-holder. |
| Transport | Defined as grasp offset | When a low point in index finger velocity had been reached after the index finger had been moved into the goal-holder area (20 mm in fronto-parallel plane from goal-holder center). |
| Fitting | Defined as transport offset | When the wrist marker exceeded a velocity of 60 mm/s (or, in disc trials with velocity continuously above 60 mm/s, exceeding a velocity low point) in the process of removing the hand from the released peg |
