# Supplementary material for: Atypical development of sequential manual motor planning and visuomotor integration in children with autism at early school-age: A longitudinal kinematic study
Source: Autism. 2025 Jan 6;29(6):1510–23. doi: 10.1177/13623613241311333 (PMC12089664; doi:10.1177/13623613241311333)
Supplement: sj-docx-2-aut-10.1177_13623613241311333 – Supplemental material for Atypical development of sequential manual motor planning and visuomotor integration in children with autism at early school-age: A longitudinal kinematic study [file sj-docx-2-aut-10.1177_13623613241311333.docx]

| Supplementary Table S2  Hypothesis 1a investigated in visual and occluded condition respectively: F*statistics and*p*-values for the main effects and specified interactions, and observed developmental patterns in each group.* | | |
| --- | --- | --- |
| Kinematic variable | Visual Condition  Fixed effects:  Main effects and specified interactions | Occluded condition  Fixed effects:  Main effects and specified interactions |
| Latency | **A: F(2, 118.3)=31.891, *p<.001***  **[**TD: A1>A2>A3; p≤.008]  [ASD: A1>A2>A3; p≤.036]  G: F(1, 29.5)=.602, *p=.444*  O: F(3, 82.8)=.665, *p=.576*  AxG: F(2, 118.3)=.254, *p=.776*  GxO: F(3, 82.8)=.733, *p=.535* | **A: F(2, 118.5)=20.360, *p<.001***  [TD: A1>A2 and A1>A3; p≤.002]  [ASD: A1>A3 and A2>A3; p<.001]  G: F(1, 28.9)=.229, *p=.588*  O: F(3, 83.9)=2.134, *p=.102*  AxG: F(2, 118.5)=2.426, *p=.093*  GxO: F(3, 83.9)=1.703, *p=.173* |
| PPV-RTG | **A: F(2, 118.1)=26.906, *p<.001***  [TD: A1<A2<A3; p<.001]  [ASD: A1<A2 and A1<A3; p≤.046]  G: F(1, 28.4)=.648, *p=.427*  O: F(3, 85.1)=2.673, *p=.052*  **AxG: F(2, 118.1)=7.778, *p<.001***  GxO: F(3, 85.1)=.770, *p=.514* | **A: F(2, 117.5)=13.305, *p<.001***  [TD: A1<A2 and A1<A3; p≤.006]  [ASD: A1<A2 and A1<A3; p≤.014]  G: F(1, 27.8)=.261, *p=.614*  O: F(3, 83.5)=2.458, *p=.069*  AxG: F(2, 117.5 )=.355, *p=.702*  GxO: F(3, 83.5)=.650, *p=.585* |
| PV-RTG | **A: F(2, 118.2)=14.818, *p<.001***  [TD: A1<A3 and A2<A3; p≤.009]  [ASD: A1<A3; p=.007]  G: F(1, 28.0)=.192, *p=.665*  **O: F(3, 82.8)=10.392, *p<.001***  AxG: F(2, 118.2 )=.357, *p=.700*  GxO: F(3, 82.8)=2.519, *p=.064* | **A: F(2, 118.7)=6.901, *p<.001***  [TD: A1<A2 and A1<A3; p≤.033]  [ASD n.s.]  G: F(1, 28.8)=.009, *p=.926*  **O: F(3, 82.6)=4.136, *p=.009***  AxG: F(2, 118.7 )=1.297, *p=.277*  GxO: F(3, 82.6)=.658, *p=.580* |
| Grip duration | **A: F(2, 116.6)=18.460, *p<.001***  [TD: A1>A2>A3; p≤.002]  [ASD: A1>A2; p=.041]  G: F(1, 29.4)=.879, *p=.356*  **O: F(3, 83.8)=4.762, *p=.004***  **AxG: F(2, 116.6)=3.664, *p=.029***  GxO: F(3, 83.8)=.159, *p=.924* | **A: F(2, 119.3)=15.815, *p<.001***  [TD: A1>A2>A3; p≤.045]  [ASD: A1>A2; p=.022]  G: F(1, 29.0)=1.926, *p=.176*  **O: F(3, 83.6)=6.850, *p<.001***  **AxG: F(2, 119.3)=5.752, *p=.004***  GxO: F(3, 83.6)=.701, *p=.554* |
| RA | **A: F(2, 59.4)=4.563, *p=.014***  [TD: A1>A3; p=.012]  [ASD n.s.]  **G: F(1, 31.1)=6.414, *p=.017***  **O: F(1, 28.2)=106.741, *p<.001***  AxG: F(2, 59.4)=1.012, *p=.370*  GxO: F(1, 28.2)=.100, *p=.754* | A: F(2, 58.5)=2.660, *p=.078*  [TD n.s.]  [ASD n.s.]  G: F(1, 30.0)=.801, *p=.378*  **O: F(1, 29.3)=70.404, *p<.001***  AxG: F(2, 58.5)=.610, *p=.547*  GxO: F(1, 29.3)=.004, *p=.949* |
| PV-Transport | **A: F(2, 115.2)=13.863, *p<.001***  [TD: A1<A2 and A1<A3; p≤.010]  [ASD: A1<A3; p=.034]  G: F(1, 30.3)=2.673, *p=.112*  **O: F(3, 86.8)=43.015, *p<.001***  AxG: F(2, 115.2)=.900, *p=.409*  **GxO: F(3, 86.8)=2.814, *p=.044*** | **A: F(2, 117.4)=17.075, *p<.001***  [TD: A1<A2 and A1<A3; p≤.004]  [ASD: A1<A3; p<.001]  G: F(1, 29.8)=.102, *p=.751*  **O: F(3, 84.6)=56.205, *p<.001***  AxG: F(2, 117.4)=.181, *p=.834*  **GxO: F(3, 84.6)=2.841, *p=.043*** |
| Fitting duration | **A: F(2, 118.3)=27.880, *p<.001***  [TD: A1>A2>A3; p≤.012]  [ASD: A1>A3 and A2>A3; p≤.006]  **G: F(1, 30.9)=11.062, *p=.002***  **O: F(3, 88.5)=109.019, *p<.001***  AxG: F(2, 118.3)=2.464, *p=.089*  GxO: F(3, 88.5)=.744, *p=.529* | **A: F(2, 118.1)=23.579, *p<.001***  [TD: A1>A2 and A1>A3; p<.001]  [ASD: A1>A3 and A2>A3; p<.001]  **G: F(1, 30.5)=12.929, *p=.001***  **O: F(3, 88.0)=123.471, *p<.001***  **AxG: F(2, 118.1)=3.620, *p=.030***  GxO: F(3, 88.0)=.814, *p=.490* |
| *Note*: PPV-RTG= Percentage time to peak velocity in reach-to-grasp phase; PV-RTG= Peak velocity in reach-to-grasp phase; RA= Residual angle; PV-Transport= Peak velocity in transport phase A= Age-level; G= Group; O= Orientation; TD= Typical development; ASD= Autism spectrum disorder; Information in brackets describes observed age-level developmental patterns in each group | | |
