# Supplementary material for: Atypical development of sequential manual motor planning and visuomotor integration in children with autism at early school-age: A longitudinal kinematic study
Source: Autism. 2025 Jan 6;29(6):1510–23. doi: 10.1177/13623613241311333 (PMC12089664; doi:10.1177/13623613241311333)
Supplement: sj-docx-3-aut-10.1177_13623613241311333 – Supplemental material for Atypical development of sequential manual motor planning and visuomotor integration in children with autism at early school-age: A longitudinal kinematic study [file sj-docx-3-aut-10.1177_13623613241311333.docx]

**Supplementary Table S3**

Hypothesis 1b investigating chaining in visual and occluded condition respectively: F*statistics and*p-*values for the main effects and the specified three-way interaction (Age-level x Group x Orientation) as well as simple effects of orientation within each level combination of the other effects shown. Chaining is expressed as evident orientation effects in respective group at each age-level.*

| Variable | Fixed effects:  Main effects and specified three-way interaction | *p* values for simple effects of orientation within each level combination of the other effects shown | | | |
| --- | --- | --- | --- | --- | --- |
|  |  | Group | A1 | A2 | A3 |
| **Visual Condition** |  |  |  |  |  |
| PPV-RTG | **A: F(2, 112.0)=26.61, *p<.001***  G: F(1, 28.4)=.649, *p=.427*  O: F(3, 87.1)=2.66, *p=.053*  AxGxO: F(17, 116.3)=1.43, *p=.135* | TD | .940 | .831 | .347 |
|  |  | ASD | .092 | .294 | .864 |
| PV-RTG | **A: F(2, 112.3)=14.68, *p<.001***  G: F(1, 28.0)=.191, *p=.665*  **O: F(3, 62.0)=8.73,** ***p<.001***  AxGxO: F(17, 102.6)=.951, *p=.518* | TD | .447 | .228 | **.003** |
|  |  |  |  |  | **[**180^◦^< disc; 90^◦^< disc] |
|  |  | ASD | .269 | .242 | .389 |
| PV-Transport | **A: F(2, 109.7)=16.22, *p<.001***  G: F(1, 30.3)=2.69, *p=.111*  **O: F(3, 66.2)=41.64, *p<.001***  **AxGxO: F(17, 100.2)=3.08, *p<.001*** | TD | **<.001** | **<.001** | **<.001** |
|  |  |  | **[**180^◦^<90^◦^; 180^◦^<0^◦^; 180^◦^<disc; 90^◦^<0^◦^; 90^◦^<disc] | **[**180^◦^<0^◦^;180^◦^<disc; 90^◦^< disc] | **[**180^◦^<0^◦^; 180^◦^<disc; 90^◦^<0^◦^; 90^◦^<disc] |
|  |  | ASD | **.026** | .131 | **<.001** |
|  |  |  | [n.s.] |  | **[**180^◦^<90^◦^; 180^◦^<0^◦^; 180^◦^<disc; 90^◦^<disc] |
| **Occluded condition** |  |  |  |  |  |
| PPV-RTG | **A: F(2, 112.0)=13.56, *p<.001***  G: F(1, 27.8)=.260, *p=.614*  O: F(3, 87.7)=1.56, *p=.204*  AxGxO: F(17, 123.6)=.729, *p=.768* | TD | .899 | .320 | .296 |
|  |  | ASD | .452 | .134 | .229 |
| PV-RTG | **A: F(2, 112.8)=6.70, *p=.002***  G: F(1, 28.8)=.009, *p=.927*  O: F(3, 59.7)=2.69, *p=.054*  AxGxO: F(17, 103.5)=.474, *p=.960* | TD | .719 | .992 | .544 |
|  |  | ASD | .954 | .957 | .129 |
| PV-Transport | **A: F(2, 112.1)=16.68, *p<.001***  G: F(1, 29.8)=.102, *p=.752*  **O: F(3, 81.6)=56.12, *p<.001***  AxGxO: F(17, 121.6)=.824, *p=.663* | TD | **<.001** | **<.001** | **<.001** |
|  |  |  | [180^◦^<0^◦^; 180^◦^<disc; 90^◦^<disc] | [180^◦^<0^◦^; 180^◦^<disc; 90^◦^<disc] | [180^◦^<0^◦^; 180^◦^<disc; 90^◦^<disc] |
|  |  | ASD | **.016** | **<.001** | **<.001** |
|  |  |  | **[**180^◦^<disc] | **[**180^◦^<disc; 0^◦^<disc] | **[**180^◦^<disc; 90^◦^<disc; 0^◦^<disc] |
| *Note*: PPV-RTG= Percentage time to peak velocity in reach-to-grasp phase; PV-RTG= Peak velocity in reach-to-grasp phase; PV-Transport= Peak velocity in transport phase A= Age-level; G= Group; O= Orientation; TD= Typical development; ASD= Autism spectrum disorder; Information in brackets describes the pattern of orientation adjustments. | | | | | |
