# Supplementary material for: Atypical development of sequential manual motor planning and visuomotor integration in children with autism at early school-age: A longitudinal kinematic study
Source: Autism. 2025 Jan 6;29(6):1510–23. doi: 10.1177/13623613241311333 (PMC12089664; doi:10.1177/13623613241311333)
Supplement: sj-docx-4-aut-10.1177_13623613241311333 – Supplemental material for Atypical development of sequential manual motor planning and visuomotor integration in children with autism at early school-age: A longitudinal kinematic study [file sj-docx-4-aut-10.1177_13623613241311333.docx]

| Supplementary Table S4  Hypothesis 2 investigating differences between conditions (visual, occluded) over time: F*statistics and*p*-values for the main effects and specified interactions including 95% confidence interval for main effects* | | |
| --- | --- | --- |
| Kinematic variable | Fixed effects:  Main effects and significant interactions | 95% Confidence interval |
| Latency difference between conditions | A: F(2, 28.2)=.168, *p=.846*  **[**TD n.s.]  [ASD n.s.]  G: F(1, 29.2)=.453, *p=.506*  AxG: F(2, 28.2)=1.585, *p=.223* | A1:.010-.128, A2:.049-.113, A3.047-.101  TD:.026-.104, ASD:.041-.128 |
| PPV-RTG difference between conditions | **A: F(2, 28.2)=6.258, *p=.006***  **[**TD: A1<A3 and A2<A3; p≤.005]  [ASD n.s.]  G: F(1, 28,0)=.417, *p=.524*  **AxG: F(2, 28.2 )=5.904, *p=*.007** | A1:1.550-1.556, A2:-1.240-1.534, A3:.727-3.176  TD:.023-2.254, ASD:-.712-1.904 |
| PV-RTG difference between conditions | A: F(2, 27.2)=3.107, *p=.061*  **[**TD n.s.]  [ASD n.s.]  **G: F(1, 27.3)=5.060, *p=.033***  AxG: F(2, 27.2)=1.909, *p=.168* | A1:13.63-43.83, A2:17.78-57.28, A3:37.22-75.20  TD:11.84-43.37, ASD:35.81-72.27 |
| Grip duration difference between conditions | A: F(2, 27.1)=1.174, *p=.324*  **[**TD n.s.]  [ASD n.s.]  **G: F(1, 26.7)=4.61, *p=.041***  AxG: F(2, 27.1)=2.541, *p=.097* | A1:.009-.043, A2: -.010-.031, A3:.013-.057  TD:.000-.026, ASD:.019-.049 |
| RA difference between conditions | A: F(2, 28.1)=.175, *p=.840*  **[**TD n.s.]  [ASD n.s.]  G: F(1, 28.9)=.464, *p=.501*  **AxG: F(2, 28.1)=3.994, *p=*.030** | A1:-2.056-6.610, A2:-2.416-9.086, A3:-.542-8.048  TD:-.293-8.861, ASD:-3.314-7.234 |
| PV-Transport difference between conditions | A: F(2, 27.6)=.105, *p=.901*  **[**TD n.s.]  [ASD n.s.]  **G: F(1, 28.2)=.7.898, *p=.009***  AxG: F(2, 27.6)=1.346, *p=.277* | A1:-2.955-48.82, A2:-3.055-59.38, A3:1.712-43.27  TD:-29.76-23.42, ASD:21.85-82.60 |
| Fitting Duration difference between conditions | A: F(2, 29.1)=.440, *p=.648*  **[**TD n.s.]  [ASD n.s.]  G: F(1, 29.7)=.578, *p=.453*  AxG: F(2, 29.1)=2.649, *p=.088* | A1:-.074-.086, A2:-.044-.089, A3:-.068-.032  TD:-.063-.041, ASD:-.041-.077 |
| *Note*: PPV-RTG= Percentage time to peak velocity in reach-to-grasp phase; PV-RTG= Peak velocity in reach-to-grasp phase; RA= Residual angle; PV-Transport= Peak velocity in transport phase; A= Age-level; G = Group; TD = Typical development; ASD = Autism spectrum disorder; Information in brackets describes observed age-level developmental patterns in each group | | |
|  | | |
